# Atlantic Monthly and Dr. Henry J. Bowditch

**Published:** 1869-01

**Authors:** 


Atlantic Monthly and Dr. Henry J. Bowditch.—The January number o this literary magazine contains among other instructive and interesting articles, a popular paper upon Consumption—its causes—its eradication. He first describes briefly its nature, speaks of its relative prevalence formerly and at the present time; says it lessens in the United States from North to South; at present kills about one-quarter who die in Massachusetts and one-sixteenth part of those dying in Louisiana. Residence on a damp soil as a cause of consumption is considered in detail, with illustrative evidence. The questions of hereditary transmission, and contagious propagation, with influence of trades and professions as causes are discussed quite in detail for a paper designed for popular reading. The whole article is instructive and will be received by the readers of the Atlantic Monthly as embodying the present views of the profession.

